# Molecular Therapeutic Targets for Glioma Angiogenesis

**DOI:** 10.1155/2010/351908

**Published:** 2010-04-18

**Authors:** Shingo Takano, Toshiharu Yamashita, Osamu Ohneda

**Affiliations:** ^1^Department of Neurosurgery, Institute of Clinical Medicine, University of Tsukuba, Tsukuba Ibaraki 305-8575, Japan; ^2^Regenerative Medicine/Stem Cell Biology, Institute of Basic Medical Science, University of Tsukuba, Tsukuba Ibaraki 305-8575, Japan

## Abstract

Due to the prominent angiogenesis that occurs in malignant glioma, antiangiogenic therapy has been attempted. There have been several molecular targets that are specific to malignant gliomas, as well as more broadly in systemic cancers. In this review, I will focus on some topics related to molecular therapeutic targets for glioma angiogenesis. First, important angiogenic factors that could be considered molecular targets are VEGF, VEGF-induced proteins on endothelial cells, tissue factor, osteopontin, *α*_v_ *β*_3_ integrin, and thymidine phosphorylase as well as endogenous inhibitors, soluble Flt1, and thrombospondin 1. Second, hypoxic areas are also decreased by metronomic CPT11 treatment as well as temozolomide. Third, glioma-derived endothelial cells that are genetically and functionally distinct from normal endothelial cells should be targeted, for example, with SDF-1 and CXCR7 chemokine. Fourth, endothelial progenitor cells (EPCs) likely contribute towards glioma angiogenesis in the brain and could be useful as a drug delivery tool. Finally, blockade of delta-like 4 (Dll4) results in a nonfunctioning vasculature and could be another important target distinct from VEGF.

## 1. Introduction

Malignant gliomas tend to be highly vascularized and contain hypoxic regions. Therefore, an antiangiogenic strategy is promising for malignant gliomas [1, 2]. In this review, I focus on some molecular therapeutic targets for glioma angiogenesis: (1) angiogenic factors, (2) hypoxia, (3) glioma-derived endothelial cells, and (4) resistance to antiangiogenic therapy. The problem of how to treat patients who fail to respond to antiangiogenic therapy remains a challenge, and the mechanisms of resistance are being studied. The potential mechanisms by which tumor cells can evade antiangiogenic therapy include upregulation of non-VEGF-mediated pathways of angiogenesis, recruitment of bone marrow-derived cells, increased pericyte coverage, and increased use of preexistent vasculature by invasion [3].

## 2. Angiogenic Growth Factors in Glioblastoma

**Figure 1:**
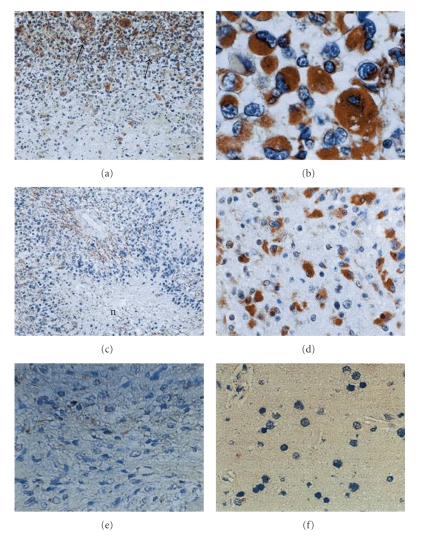
VEGF localization in gliomas. (a)–(c) Glioblastoma. VEGF localizes in the cytoplasm of the tumor cells and tumor capillary around the necrosis and the tumor periphery. (d) Anaplastic astroctytoma. (e) Diffuse astrocytoma. (f) Normal brain. Original magnification (a, c) ×50, (b, d, e, f) ×200.

**Figure 2:**
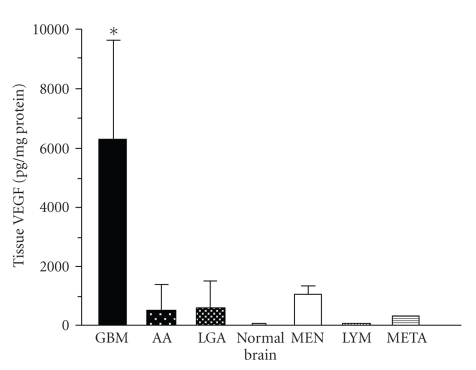
VEGF concentration in the various brain tumors.

Vascular endothelial growth factor (VEGF) is a major angiogenic factor in glioblastoma [4]. VEGF is localized within tumor cytoplasm and endothelium (Figure 1). VEGF is predominantly located in the perinecrotic area, which is referred to as the pseudopallisading area and appears to be hypoxic. By contrast, there are few VEGF positive cells in low-grade astrocytomas and no VEGF positive cells in the normal brain. We initially demonstrated increased expression of VEGF in malignant glioma tissues, with both ELISA (Figure 2) and immunohistochemistry. We also demonstrated high VEGF protein concentrations in the cyst fluid from glioma, but VEGF was not detectable in the serum.

**Figure 3:**
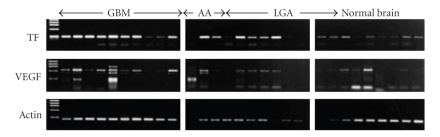
Tissue factor and VEGF mRNA expression in human glioma samples. Tissue factor expression was frequent and highly observed in glioblastomas associating with VEGF expression.

**Figure 4:**
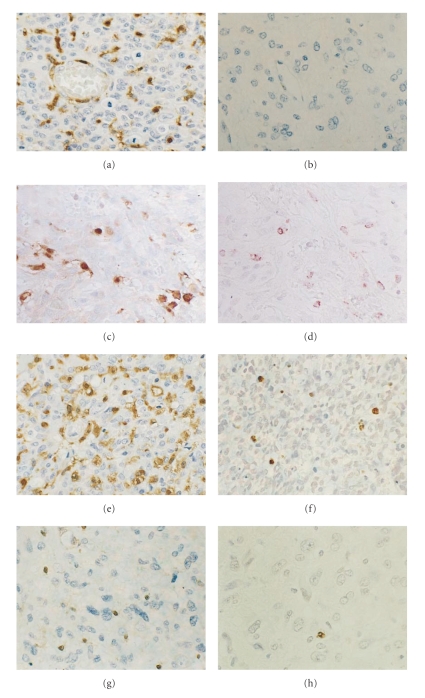
Thymidine phosphorylase immunohistochemistry in human gliomas. Glioblastoma shows intense immunoreaction for thymidine phosphorylase both in tumor and endothelial cells (a). Diffuse astrocytoma shows no expression (b). Some of the tymidine phosphorylase positive cells (c) are macrophages ((d) serial section of (c)). Thymidine phosphorylase positive glioblastoma (e) reveals a high apoptotic index ((f) serial section of (e)), while Thymidine phosphorylase negative glioblastoma (g) reveals a low apoptotic index ((h) serial section of (g)). Original magnification ×200.

VEGF-related angiogenic factors have been also clearly demonstrated in glioma tissues by RT-PCR and immunohistochemistry. Tissue factor is highly expressed in malignant gliomas associated with VEGF expression (Figure 3) [5]. Also, osteopontin and *α*_v_ *β*_3_ integrin, which are also induced by VEGF in endothelium, are predominantly expressed in tumor endothelium. Another angiogenic factor, thymidine phosphorylase, is also only expressed in malignant gliomas, but not in low-grade glioma or normal brain (Figure 4) [6]. 

**Figure 5:**
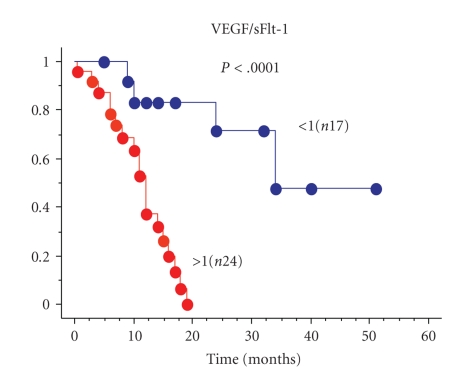
Malignant glioma survival by VEGF/sFlt-1 ratio.

**Figure 6:**
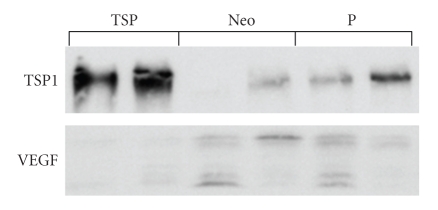
Characterization of thrombospondin-1 transfected U87. Thrombospondin-1 expression was markedly elevated in the transfectant (TSP) compared to vector alone (Neo) and parent U87 (P). Also VEGF expression was decreased in transfectant (TSP).

**Figure 7:**
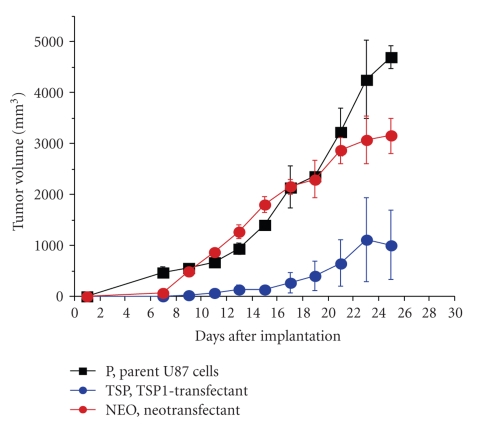
Inhibition of glioma growth by thrombospondin-1 transfection. The glioma growth is significantly inhibited by thrombospondin-1 transfectant (TSP-1 transfectant) compared to parent U87 and vector alone (Neotransfectant).

Endogenous angiogenesis inhibitors are important molecules in the delicate balance of angiogenic potential in tumors. The soluble form of the VEGF receptor 1 (sFlt-1) is a measurable, potent, and specific VEGF inhibitor. The concentrations of sFlt-1 and VEGF have been measured in glioma tissues by ELISA. A VEGF/sFlt-1 ratio greater than 1 is a worse prognostic factor in glioblastomas (Figure 5) [7]. The significance of the VEGF/sFlt-1 ratio as a prognostic factor is greater than for the VEGF concentration alone, suggesting that the angiogenic balance between angiogenic factor and its inhibitor is important in tumor angiogenesis. Experimentally, transfection of human glioma cells with sFlt-1 demonstrated low expression of VEGF mRNA compared to transfection with an empty vector. The tumor growth of these sFlt-1 transfectants was inhibited, but the inhibitory activity was limited [7]. Another endogenous angiogenic inhibitor, thrombospondin1 (TSP1), was introduced into human U87 glioma cells by transfection (Figure 6). The glioma growth of the TSP1 transfectant was significantly inhibited compared to those of parent and vector-alone transfectants (Figure 7) [7]. These clinical and experimental data support the importance of angiogenic balance as a key factor in antiangiogenic therapy. This is likely because these endogenous angiogenesis inhibitors are upregulated in malignant gliomas, as a consequence of the upregulation of angiogenic factors.

**Figure 8:**
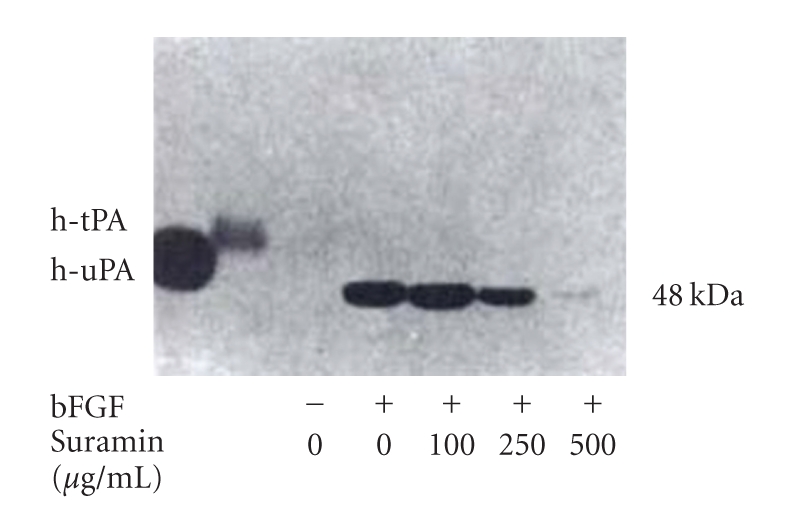
Suramin inhibition of bFGF induced endothelial cell urokinase type plasminogen activator activity on gelatin zymogram.

**Figure 9:**
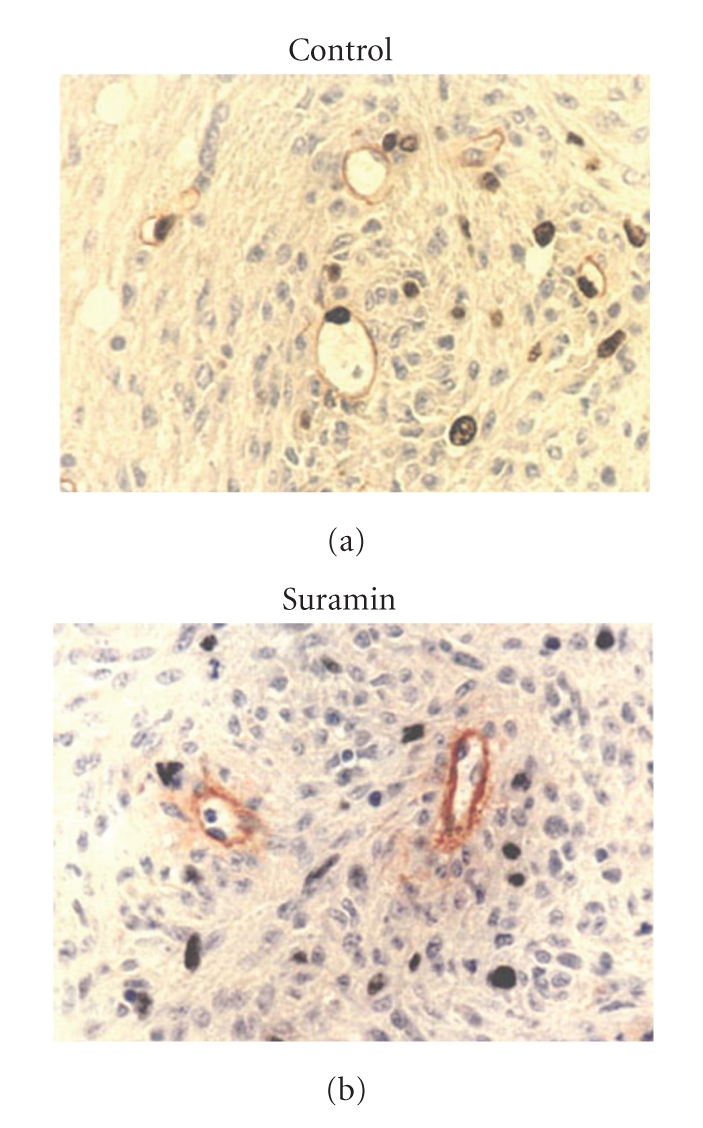
Suramin inhibition of tumor endothelial cell Ki67 labeling.

The general growth factor receptor inhibitor, suramin, was investigated for its antiangiogenic action [8]. Suramin inhibited physiologic angiogenesis in a dose-dependent manner, based on the chorioallantoic membrane (CAM) assay (Table 1). Suramin inhibited the bFGF-induced endothelial expression of urokinase-type plasminogen-activator (uPA) using gelatin zymogram (Figure 8). uPA is closely related to the initiating step of angiogenesis, degradation of the extracellular matrix. Using a rat intracranial C6 glioma model, suramin inhibited Ki67 labeling of the tumor endothelium (Figure 9) [9]. All of these data suggest that suramin can inhibit physiological and tumor angiogenesis at multiple levels. 

**Figure 10:**
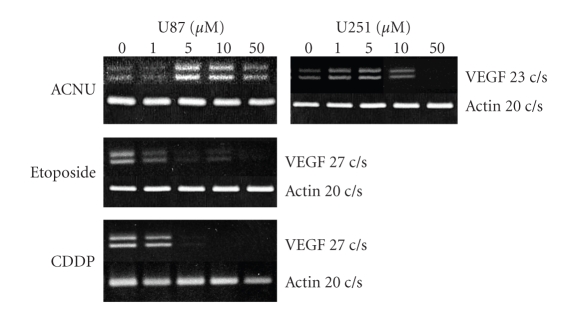
Elevation of VEGF mRNA expression by ACNU treatment, but not etoposide and CDDP treatment, in human glioma cells (U87 and U251).

**Figure 11:**
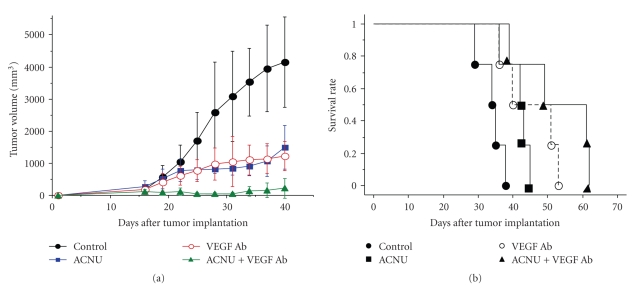
Glioma growth inhibition by VEGF antibody, ACNU, and the combination of both treatments with U87 subcutaneous (a) and intracranial (b) model.

**Figure 12:**
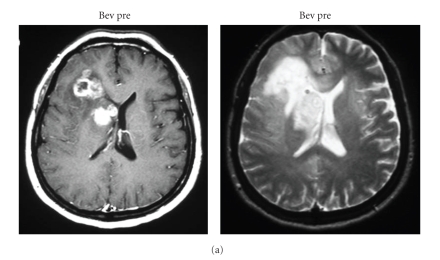

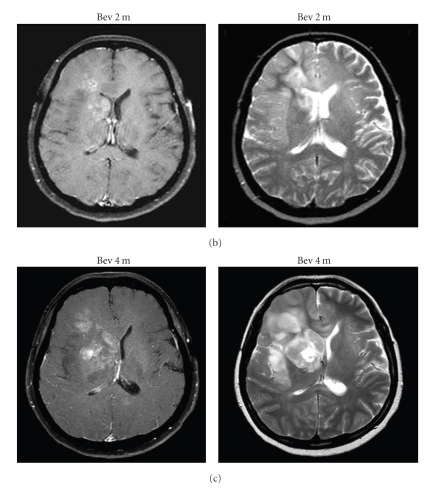
Clinical course of a case with recurrent malignant glioma with bevacizumab and CPT11 treatment. After 3 cycles enhanced tumor and perifocal edema is markedly diminished (Bev 2 m). However, after 6 cycles T2 high intense tumors regrow with minimal enhancement (Bev 4 m).

Recently, the anti-VEGF antibody, bavacizumab, has been used in the treatment of glioblastoma [10]. The growth inhibitory effect is dramatic, especially when determined with MRI enhancement and MRI perfusion study [11]. Interestingly, ACNU (1-(4-amino-2-methyl-5-pyrimidinyl)-methyl-3-(2-cholroethyl)-3-nitrosourea hydrochloride) chemotherapeutic agents resulted in upregulation of VEGF mRNA in glioma cells (Figure 10) [12]. A similar effect on upregulation of VEGF has been demonstrated by irradiation of glioma cells. Thus, the combination of VEGF antagonism, that is, VEGF antibody in the initial glioma therapy, is a reasonable strategy in ACNU chemotherapy and in radiation therapy (Figure 11). The VEGF antibody is attractive for attacking tumor stem cells, as a new strategy to combat glioblastomas, because the VEGF antibody could inhibit maintenance of glioma stem cells by destroying glioma vascular niche [13, 14], in contrast to the effects of radiation or other chemotherapeutics. However, the antiangiogenic antiglioma effect is transient (Figure 12). Congruent with the results obtained in orthotropic mouse models of GBM, four recent clinical studies have implicated proinvasive adaptation in humans, as observed by MRI, in a subset of GBM patients who developed multifocal or diffuse recurrence of the tumor during a course of anti-VEGF therapy with bevacizumab, as in our case [15–17]. Thus, there is a clear need for alternative strategies after bevacizumab failure.

## 3. Hypoxia in Gliomas

**Figure 13:**
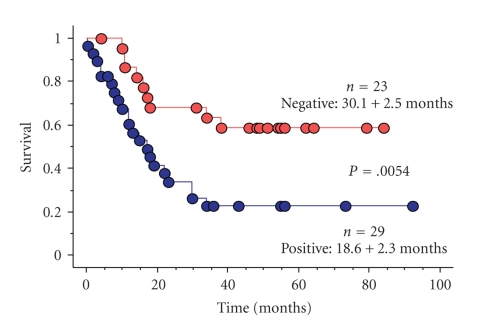
Malignant glioma survival with hypoxia inducible factor 1*α* (HIF-1*α*) expression on immunohistochemistry. HIF-1*α* expression is a negative prognostic factor.

**Figure 14:**
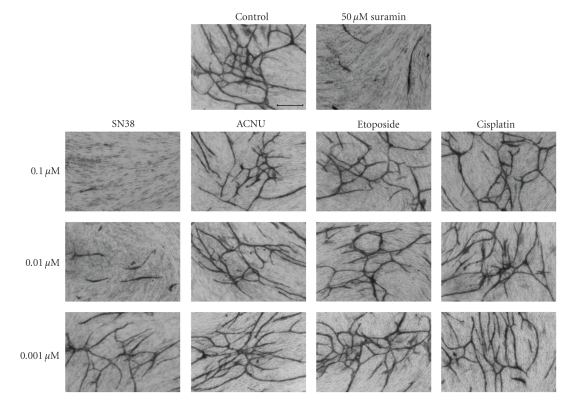
Antiangiogenic effect of SN38, active metabolite of CPT11. Low dose of SN38 (0.01 and 0.1 *μ*M) inhibited tube formation of HUVEC.

**Figure 15:**
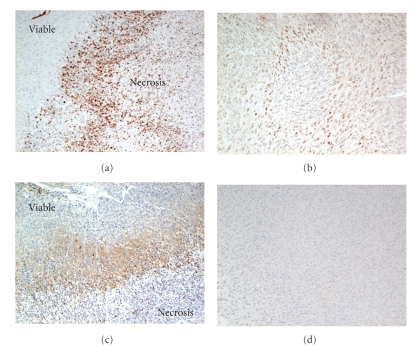
Hypoxia inducible factor 1*α* expression and hypoxic area with (b, d) and without (a, c) metronomic CPT11 treatment. HIF-1*α* expression and hypoxic area around the necrosis of glioma tissue decreased with treatment.

One of the mechanisms of resistance to angiogenic treatment is the presence of hypoxic regions in glioma tissues. Hypoxia-inducible factor 1*α* (HIF1*α*) is induced by hypoxia and is upstream of VEGF mRNA expression. Immunohistochemical expression of HIF-1*α* clearly correlated with the degree of glioma malignancies and predicted survival among patients with malignant gliomas (Figure 13) and the degree of necrosis on MRI (data not shown). Therefore, HIF-1*α* has been the focus of antiangiogenic treatment [18]. Downregulation of HIF-1*α* in glioma cells using siRNA resulted in growth inhibition and an angiosuppressive effect on glioma growth (unpublished data). Metronomic chemotherapy is a promising strategy to overcome resistance to antiangiogenic treatment [19]. We demonstrated that SN38, the active metabolite of CPT11, exhibited an antiangiogenic effect (Figure 14). SN38 inhibited HIF-1*α* and VEGF mRNA and protein expression of glioma cells in a dose- and time-dependent manner [20]. Metronomic CPT11 treatment of gliomas exhibited growth inhibitory effects without systemic toxicity, that is, through comparison of body weight loss that was not observed by conventional CPT11 treatment. Tumor tissues treated with metronomic CPT11 exhibited decreased expression of HIF-1*α* protein and pimonidazole expression, which were indicative of areas of hypoxia by immunohistochemistry (Figure 15). A recent advance in glioma chemotherapy is the discovery of temozolomide. Temozolomide is a powerful chemotherapeutic agent that prolongs overall survival of initial glioblastoma by up to 2.5 months [21]. More recently, the feasibility of bevacizumab with radiation therapy and temozolomide in newly diagnosed high-grade gliomas has been reported [22, 23]. Interestingly, temozolomide has an inhibitory effect on HIF-1*α* expression and endothelial cell tube formation [24]. The metronomic temozolomide treatment is reasonable and clinical results have been demonstrated [25, 26].

## 4. Glioma-Derived Endothelial Cells

**Figure 16:**
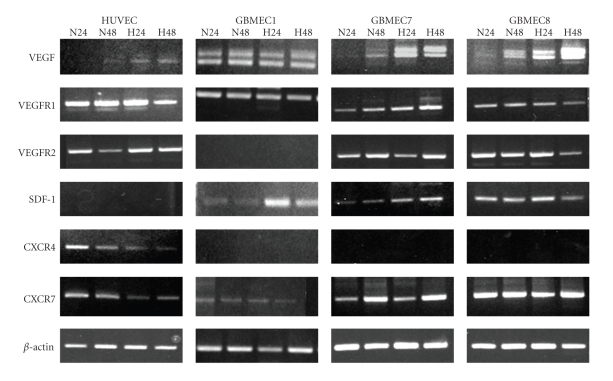
Angiogenic factor and chemokine expression in HUVEC and glioblastoma derived endothelial cells (GBMECs). GBMECs show high expression of VEGF, SDF-1, and CXCR7 compared to HUVEC and no expression of CXCR4.

Many studies focusing on tumor angiogenesis and endothelial biology are based on established normal cells lines that is, human umbilical vein endothelial cells (HUVECs). Whether or not tumor endothelial cells and normal endothelial cells are genetically and functionally identical remains controversial. Comparisons between tumor-derived and normal ECs have been made for a variety of systemic tumors [27]. They have shown that tumor endothelium exhibits a phenotype of activated ECs, as reflected in the high expression of angiogenic molecules, that is, VEGFR, the angiopoietin receptor Tie2, and the adhesion molecules ICAM-1, E-selectin, and CD44. In recent publications, researchers have suggested that the tumor-associated ECs derived from GBM tissues have different phenotypic and functional properties compared to normal ECs [28, 29]; these differences may result in less effective antiangiogenic therapy if the target molecules are only expressed in normal blood vessels. Moreover, these publications have not mentioned the potential for interactions between tumor cells and tumor-derived endothelial cells. We isolated tumor endothelial cells from human glioblastoma samples using flow cytometry, cultured them, and analyzed the genetic differences between these cell types and HUVEC regarding the mRNA and protein expression of angiogenic factors and chemokines. Glioblastoma-derived endothelial cells (GBMECs) exhibited high expression of VEGF, SDF-1, and CXCR7 mRNA compared to HUVEC, and GBMECs exhibited no expression of CXCR4 mRNA (Figure 16, unpublished data). We are now investigating functional differences between GBMECs and HEUVEC as well as the interaction between GBMECs and glioma cells using a coculture system. To obtain successful results with antiangiogenic therapy, tumor endothelial cells should be targeted in the future.

## 5. The Role of Endothelial Progenitor Cells on Tumor Angiogenesis

**Figure 17:**
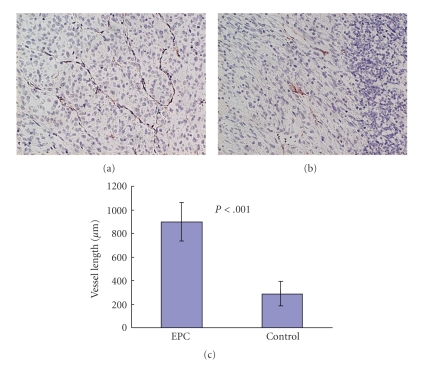
Rich vascular network with EPC-injected glioma. The vessel length of EPC-injected tumor (a) is significantly longer than those of control (b).

**Figure 18:**
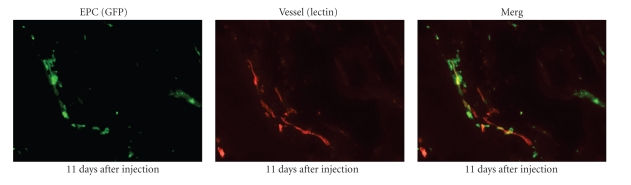
EPC homing to glioma vasculature. 11 days after injection of GFP labeled EPCs, EPCs localize lectin labeled glioma vasculature.

Another important mechanism of resistance to antiangiogenic treatment is related to EPCs. EPCs are introduced into tumor angiogenesis by tumor stimuli from the bone marrow. We investigated the role of EPCs on glioma angiogenesis. C6 glioma cells (5 × 10^6^ cells) were stereotactically implanted into the brain. After 7 days, EPCs (3 × 10^5^ cells) that were harvested from umbilical cord blood [30] were intravenously injected via the tail vein. Seven days after the EPC injection, the rats were sacrificed and the C6 gliomas in the brain were fixed and stained with CD31. EPC-injected C6 glioma demonstrated a large narrow vascular network. The vessel length is significantly longer than EPCs in an uninjected tumor (Figure 17). Fluoroscopy demonstrated that GFP-labeled EPCs localized along with lectin-labeled tumor vessels (Figure 18). This result suggests that EPC could induce homing to the glioma vasculature and that this characteristic of tumor vasculature homing is useful when considering EPCs as drug delivery tools. If the EPCs contain angiogenesis inhibitors, the angiogenesis inhibitor is automatically delivered to the tumor vasculature. To date, this new strategy has not been published.

## 6. Molecular Targets of Glioma Angiogenesis in Future

 Finally, the question remains, what are the current possible target molecules for glioma angiogenesis? Norden et al. [31] reported some molecules other than VEGF. Among them, delta-like 4 (Dll4) remains promising, because the mechanisms of angiosuppression are quite different to those of VEGF and the role of Dll4 is reciprocal to VEGF [32, 33]. Remarkably, Dll4 and VEGF are the only known genes for which loss of a single allele results in embryonic lethality due to failure to form a functional vasculature. Dll4 is exclusively expressed by endothelial cells; therefore, this ligand is a potential therapeutic target. Although blocking Dll4 appears to promote angiogenesis, the neovasculature is functionally abnormal and it cannot support tumor cell survival [34]. Preclinical studies have shown that blockade of Dll4 was effective in inhibiting the growth of tumors that are resistant to VEGF inhibition [32]. Whereas most of current antiangiogenesis approaches act through the reduction or elimination of tumor blood vessels, Dll4 blockade results in the formation of a nonfunctional vasculature that is unable to support tumor growth. This paradoxical strategy for targeting tumors will be the focus of intense research for years ahead.

Furthermore, the expression of recombinant toxic proteins that specifically target tumor endothelium appears to be promising [35]. Fusion proteins directed against urokinase-type plasminogen-activator receptor (uPAR) may be appropriate for targeting endothelial cells in the tumor vasculature compared with normal endothelium, as uPAR may be preferentially expressed in proliferating endothelium. The efficacy of protein DTAT13 that was synthesized to target uPAR on the neovasculature and uPAR- and interleukin-13-expressing glioblastoma cells has been demonstrated on glioma growth in vitro and in vivo [36].

## 7. Conclusion

 Anti-angiogenesis therapy for malignant gliomas is promising by not only inhibiting angiogenesis but also through alteration of the tumor microenvironment, that is, the tumor vascular niche. Moreover, various combinations of strategies including the development of new molecular targets have been investigated. Overcoming resistance to antiangiogenic therapy with minimal side effects should be considered.

## Figures and Tables

**Table 1:** Inhibition of angiogenesis in the chick chorioallantoic membrane assay.

Suramin (*μ*g/disk)	Embryos evaluated (positive/total)	% of inhibition
0	0/10	0
30	0/7	0
250	2/8	25
500	5/10	50
1000	12/17	71
1500	7/7	100
